# Improved Data Reporting in Alzheimer's Publications with Layered Digital Media ‐ Schol‐AR

**DOI:** 10.1002/alz70855_100522

**Published:** 2025-12-23

**Authors:** Tyler Ard, Michael S Bienkowski, Sook‐Lei Liew, Arthur W. Toga

**Affiliations:** ^1^ Stevens Neuroimaging and Informatics Institute, University of Southern California, Los Angeles, CA, USA; ^2^ Stevens Neuroimaging and Informatics Institute, Keck School of Medicine, University of Southern California, Los Angeles, CA, USA; ^3^ Neural Plasticity and Neurorehabilitation Laboratory, University of Southern California, Los Angeles, Los Angeles, CA, USA; ^4^ Laboratory of Neuro Imaging, Stevens Neuroimaging and Informatics Institute, Keck School of Medicine, University of Southern California, Los Angeles, CA, USA

## Abstract

**Background:**

Alzheimer's research spans multiple disciplines and relies on diverse data types, from brain scans to microscopic images. Traditional scientific publications often struggle to effectively convey this complex information, leading to incomplete data presentation and potential misunderstanding among readers.

**Method:**

We present the evolution of our 'Schol‐AR' system, an innovative platform that seamlessly integrates interactive digital content into scientific figures and manuscripts. This advanced system has gained significant traction and has been widely adopted by numerous prestigious scientific publishers and journals that communicate Alzheimer's disease research. The scope of 'Schol‐AR' spans numerous types of Alzheimer's studies, from cellular‐level investigations to complex neuroimaging analyses. By transforming conventional 2D static figures into dynamic, interactive visualizations, our approach dramatically enhances the accessibility and comprehensibility of Alzheimer's research data. This technological leap allows researchers, clinicians, and students to engage with digital interactive data inside manuscripts, fostering deeper understanding and potentially accelerating progress in the field.

**Result:**

The adoption of our data layering technique has seen a significant surge in adoption for research communication. By providing intuitive, interactive access to layered data, researchers can now explore intricate Alzheimer's‐related information in context, fostering deeper insights from more comprehensive reporting. This enhanced method of data presentation improves the clarity and impact of research findings and facilitates knowledge dissemination within the scientific community, potentially accelerating advancements in Alzheimer's disease research.

**Conclusion:**

Leveraging new technologies, we can revolutionize how Alzheimer's research is communicated, effectively bridging the gap between the digital nature of modern scientific data and the constraints of traditional journal article formats.

